# Activin A promoted the anti‐tumor effect of ActRIIA ^high^ CD8 ^+^ T cells in mouse hepatoma

**DOI:** 10.1002/cam4.70147

**Published:** 2024-12-27

**Authors:** Liangchang Hu, Yu Zhao, Xuguang Zhang, Chunhui Ma

**Affiliations:** ^1^ Department of Oncology Affiliated Hospital of Shandong Second Medical University Weifang China; ^2^ School of Medical Laboratory Shandong Second Medical University Weifang China; ^3^ Department of Clinical Laboratory Affiliated Hospital of Shandong Second Medical University Weifang China; ^4^ Clinical Research Center Affiliated Hospital of Shandong Second Medical University Weifang China

**Keywords:** activin A, CD8^+^ T cells, hepatoma, immune regulation, tumor immunity

## Abstract

**Background:**

Activin A, a noteworthy member of the TGF‐β superfamily. Activin A can regulate the biological functions of various immune cells, such as macrophages, neutrophils, NK cells, etc. The purpose of this study is to investigate the regulatory effect and related mechanisms of activin A on CD8^+^ T cells.

**Methods:**

This study established a mouse subcutaneous transplantation model of hepatoma to explore whether activin A has a regulatory effect of CD8^+^ T cells. And reveal the biological characteristics of ActRIIA high CD8^+^ T cells.

**Results:**

We discovered that CD8^+^ T cells express ActRIIA, ActRIIB, and Smad2,3,4. Unlike high‐dose activin A, low‐dose activin A increased the content of CD8^+^ T cells while also suppressing tumor growth. In addition, low‐dose activin A promoted expression of perforin, granzyme B, CD69, CD11c, CD49b, Ki67 and ActRIIA in CD8^+^ T cells. And the results indicated that high‐dose activin A promotes the expression of PPM1A in T cells, while low‐dose activin A has no effect on these cells. The tumor contained more ActRIIA high CD8^+^ T cells compared to the peripheral blood and spleen. ActRIIA high CD8^+^ T cells highly express cytotoxic molecules and cytokines. And ActRIIA high CD8^+^ T cells not only expressed high levels of activation/co‐stimulatory markers CD69, CD278, and CD28, but also high levels of inhibitory markers CD366 and KLRG1. Different chemokine receptors displayed varying levels of expression in ActRIIA high CD8^+^ T cells. Additionally, these cells have a high proliferative potential. Finally, overexpression of Mettl3 inhibited the expression of ActRIIA in CD8^+^ T cells.

**Conclusions:**

This study demonstrated that low‐dose activin A enhanced the anti‐tumor effect of CD8^+^ T cells, and ActRIIA high CD8^+^ T cells actively contribute to tumor immunity.

## 1 INTRODUCTION

Activin A, part of the TGF‐β superfamily, encourages the production and release of pituitary follicle stimulating hormone (FSH) with 95% species homology.^1^, ^2^ We've found three distinct types of activins only activin A, activin B and activin AB have biological activities, and the biological activities of the three are similar, but activin A has the most extensive tissue distribution and the strongest function.^3^ Activin interacts with the activin type II receptor (ActR II), subsequently engaging the activin type I receptor (ActRI). Activated ActRI further promotes Smad2 and Smad3 phosphorylation. After phosphorylated Smad2/3 is separated from the receptor, it collaborates with Smad4 to form a complex that enters the nucleus, stimulating transcription of target genes.^4^ More and more evidence shows that activin A is instrumental in our body's biological processes. Activin A not only regulates DNA synthesis and growth of hepatocytes, but also regulates the reconstruction of liver structure after partial hepatectomy.^5^ The concentration of activin A in the peripheral blood of patients with liver diseases increases, including viral hepatitis, alcoholic cirrhosis, and liver cancer.^5^ In addition, activin A regulates the biological functions of various immunocyte, including natural killer cells, neutrophils, macrophages, and lymphocytse.^6^

CD8^+^ T cells play a vital role in our adaptive immunity.^7^ Upon specific antigen stimulation, these cells proliferate, maturing into potent cytotoxic T lymphocytes capable of discerning and eliminating targeted cells.^8^ CD8^+^ T cells lysis target cells by secreting cytotoxic particles. In addition, CD8^+^ T cells can secrete cytokines such as IFN‐γ, IL‐2, and IL‐10 to moderate the immune response.^9^, ^10^ Studies have shown that when activin A reaches 34.7 ng/mL in mice, it can suppress CD8^+^ T cell infiltration in tumors, but does not affect cytotoxicity, proliferation activity, and the expression of a variety of CD8^+^ T cells functional molecules.^11^ Activin A concentration fluctuates across diverse physiological and pathological states is quite different. For example, metastatic liver cancer of unidentified origin exhibits 14.1 ng/mL activin A, and cholangiocarcinoma elucidates a level of 32 ng/mL.^12^, ^13^ Moreover, activin A levels in peripheral blood tend to escalate with pregnancy progression, and reached 18.57 ng/mL in late trimester of pregnancy.^12^ However, the concentration level of activin A in healthy individuals and patients with conditions such as hypertension, hyperthyroidism, adrenal sebaceous adenoma, cirrhosis and pituitary adenoma, about 1–2 ng/mL.^12^ In terms of its effect on CD8^+^ T cells, there are limited studies on the influence of low‐dose activin A. Therefore, we established a mouse subcutaneous transplantation model of hepatoma to investigate the potential different regulatory roles of low‐dose Activin A on CD8^+^ T cells.

## 2 RESULTS

### 2.1 Expression of activin receptors and downstream signaling molecules in mouse CD8 ^+^ T cells

**FIGURE 1:**
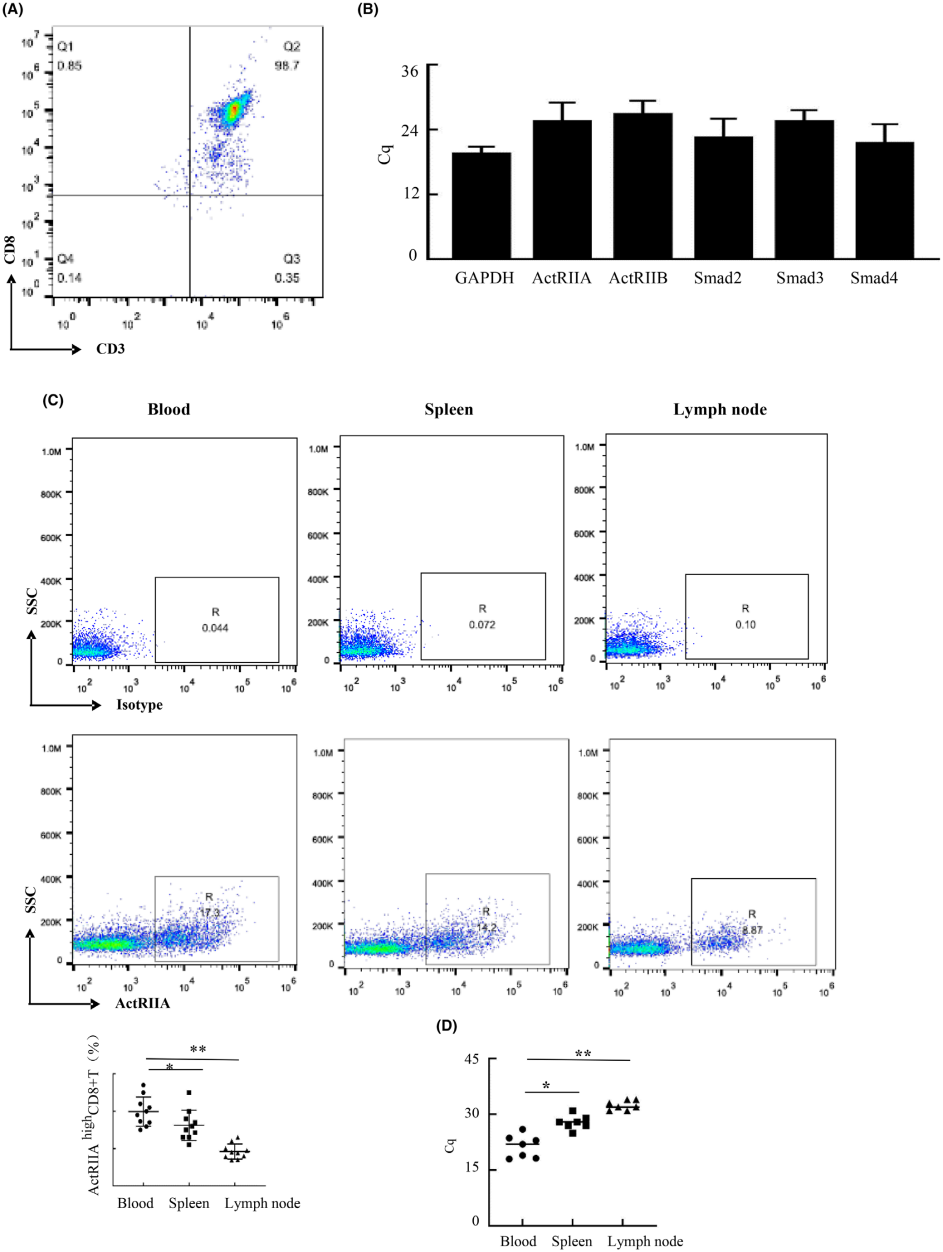
The expression of signaling molecules in the activin signaling pathway in CD8^+^ T cells. (A) Quality of isolated T cells was confirmed via anti‐CD3 and anti‐CD8 antibody staining. (B) Quantitative RT‐PCR identified ActRIIA, ActRIIB, Smad2, 3,4 in mouse CD8^+^ T cells. (C) Verified ActRIIA expression on CD8^+^ T cells by flow cytometry. (D) The expression of ActRIIA in CD8^+^ T cells from peripheral blood, spleen, and lymph nodes of mice was detected by RT‐PCR. Data shown as mean ± standard. *p < 0.05, **p < 0.01, compared with blood.

The CD8^+^ T cells in the mouse spleen were successfully enriched using magnetic beads. Our flow cytometry findings revealed that the percentage of CD3 and CD8 positive cells in enriched mouse spleen cells was 98.7%, meeting the research requirements (Figure 1A). Additionally, intriguing qRT‐PCR results indicated that both ActRIIA and ActRIIB mRNA, as well as the downstream signaling molecules Smad2, Smad3, and Smad4 mRNA, were expressed in these CD8^+^ T cells (Figure 1B). Furthermore, flow cytometry and qRT‐PCR data revealed that ActRIIA was expressed in CD8^+^ T cells (Figure 1C,D).

### 2.2 Regulation of tumor growth by activin A

**FIGURE 2:**
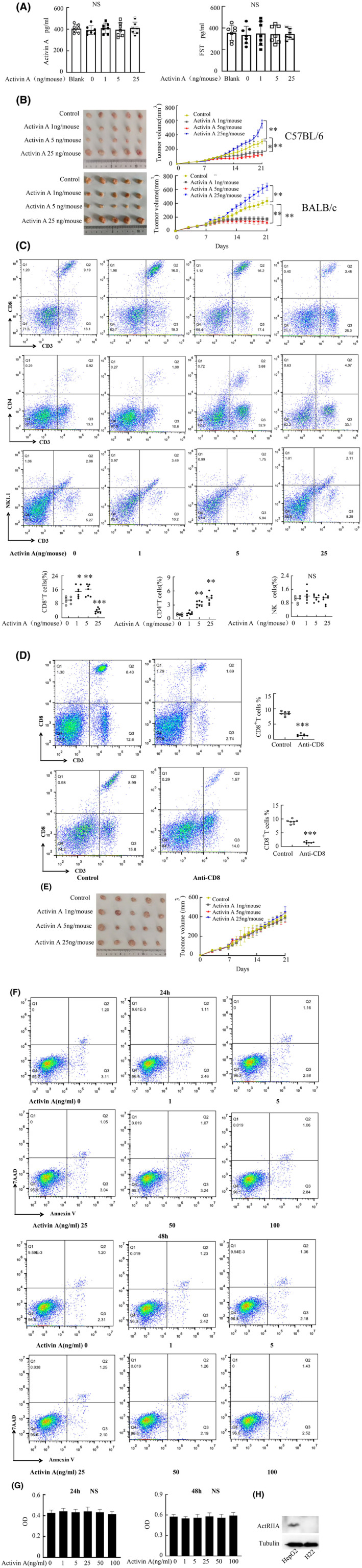
Effect of activin A on tumor growth. (A) ELISA was used to detect the activin A and FST concentrations in plasma of mice. (B) The tumor growth status of mice after intratumoral injection of activin A. (C) Regulation of intratumoral immunocyte by activin A was detected by flow cytometry. (D, E) Anti‐CD8 (10 mg/kg) initiated from Day 6, followed by biweekly administrations until study’s termination. (F) Activin A was added to HCC cultures at varying concentrations for 24 and 48 h, subsequent flow cytometry analysis measured apoptotic rates. (G) Activin A at varying concentrations was added to HCC cultures for 24 and 48 h, CCK8 was employed to assess tumor cell proliferation. (H) The expression of ActRIIA in HepG2 and H22 was examined by Western blotting. Data shown as mean ± standard.*p < 0.05, **p < 0.01, ***p < 0.001 compared with control group; NS, not significant.

A mouse subcutaneous transplantation model of hepatoma with H22 cells was constructed to detect the influence of varying concentrations of activin A on tumor growth. Firstly, the plasma activin A and FST (follistatin, FST) levels of mice were detected by ELISA at the end of the experiment. The findings indicated that there was no distinction in activin A and FST levels among healthy mice, model mice, and experimental mice (Figure 2A). The study further showed that 1 and 5 ng/mL activin A inhibited tumor growth in BALB/c and C57BL/6 mice, while 25 ng/mL activin A has the opposite effect (Figure 2B). And our research found that the survival of liver cancer patients with high serum levels of activin A is shorter than those with low serum levels of activin A (Figure S1). CD8^+^ T cells play a crucial role in the body's anti‐tumor immune response, this study will proceed to detect the content of CD8^+^ T cells in tumors, gating strategies of CD8^+^ T cells is shown in the Figure S2. Flow cytometry results indicated that 1 and 5 ng/mL activin A could increase the content of CD8^+^ T cells in tumors compared to the control group. However, when activin A concentration reached 25 ng/mL, CD8^+^ T cell content in tumors decreased. Moreover, activin A concentration‐dependently amplified the amount of CD4^+^ T cells in the tumor, without significantly affecting NK cells (Figure 2C). We observed that the effect of activin A was abrogated when CD8^+^ T cells were eliminated (Figure 2D,E). To investigate whether activin A has a direct effect on apoptosis and proliferation of H22 cells. H22 cells were exposed to varying concentrations of activin A for 24 and 48 h, followed by flow cytometry to measure apoptosis. Results revealed that diverse concentrations of activin A had minimal influence on H22 cell apoptosis (Figure 2F). CCK8 was used to detect the effect of activin A on the proliferation of H22 cells. The results displayed that different concentrations of activin A had no impact on proliferation of H22 cells (Figure 2G). Moreover, the results of Western blotting revealed that H22 did not express ActRIIA (Figure 2H).

### 2.3 Neutrophilizing effects of follistatin on low‐dose activin A on tumor growth

**FIGURE 3:**
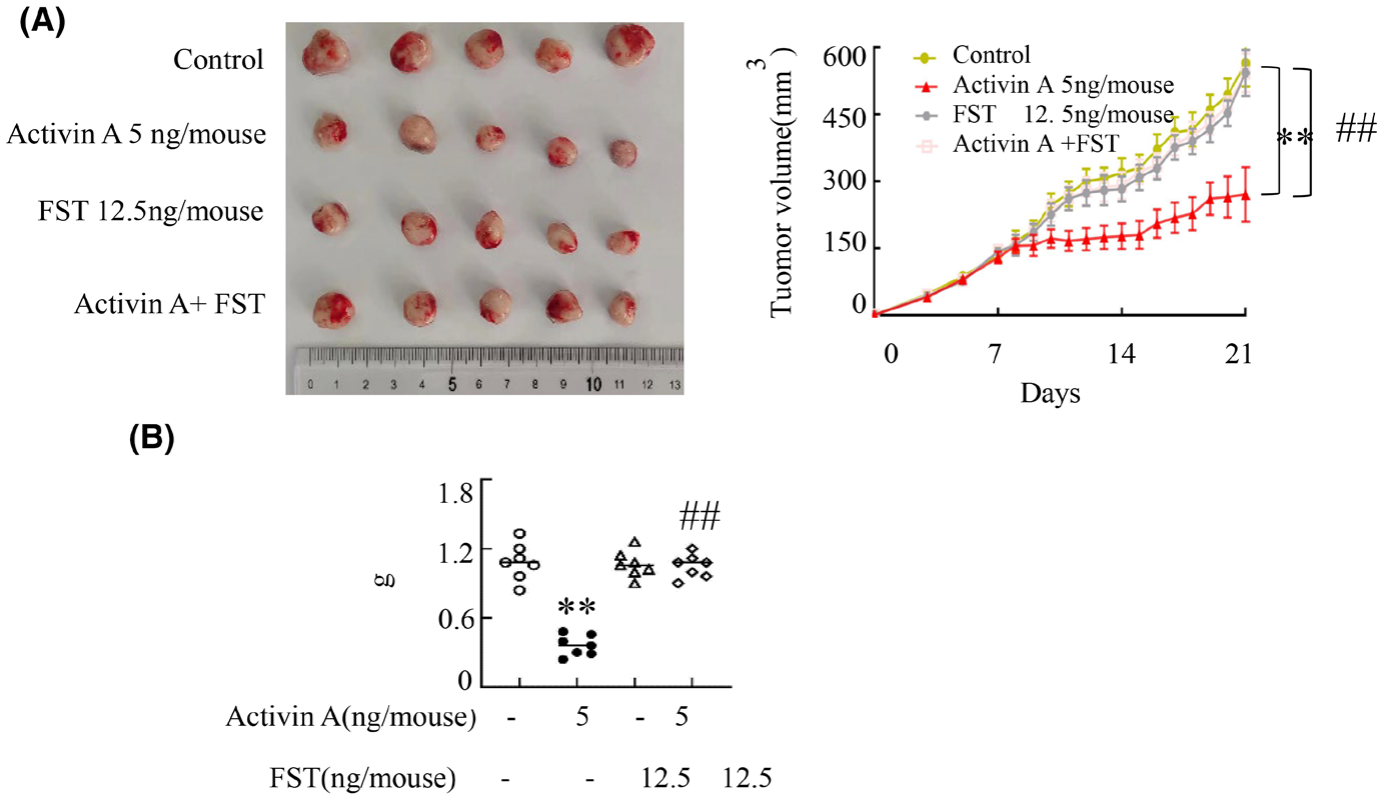
Activin A’s impact on tumor development was negated by FST treatment. Mice bearing tumors received daily intratumoral injections of either activin A or FST over a span of 14 days. Changes in tumor size (A) and weight (B) were recorded. Data shown as mean ± standard. **p < 0.01, compared with control group; ^##^ *p* < 0.01, compared with activin A group.

Neutralizing effects of follistatin (FST) toward activin A were observed via its impediment of activin A interaction with ActRIIA.^14^ Based on initial research findings, we utilized 5 ng/mouse or 5 ng/mL activin A as low dose for subsequent trials. Consequently, FST was employed to counteract the biological impact of activin A. Results indicated a significant reduction in the inhibitory influence of activin A on tumor progression (Figure 3A,B), further suggesting that low‐dose of activin A could inhibit tumor growth.

### 2.4 Effect of activin A on CD8 ^+^ T cell killing capacity

**FIGURE 4:**
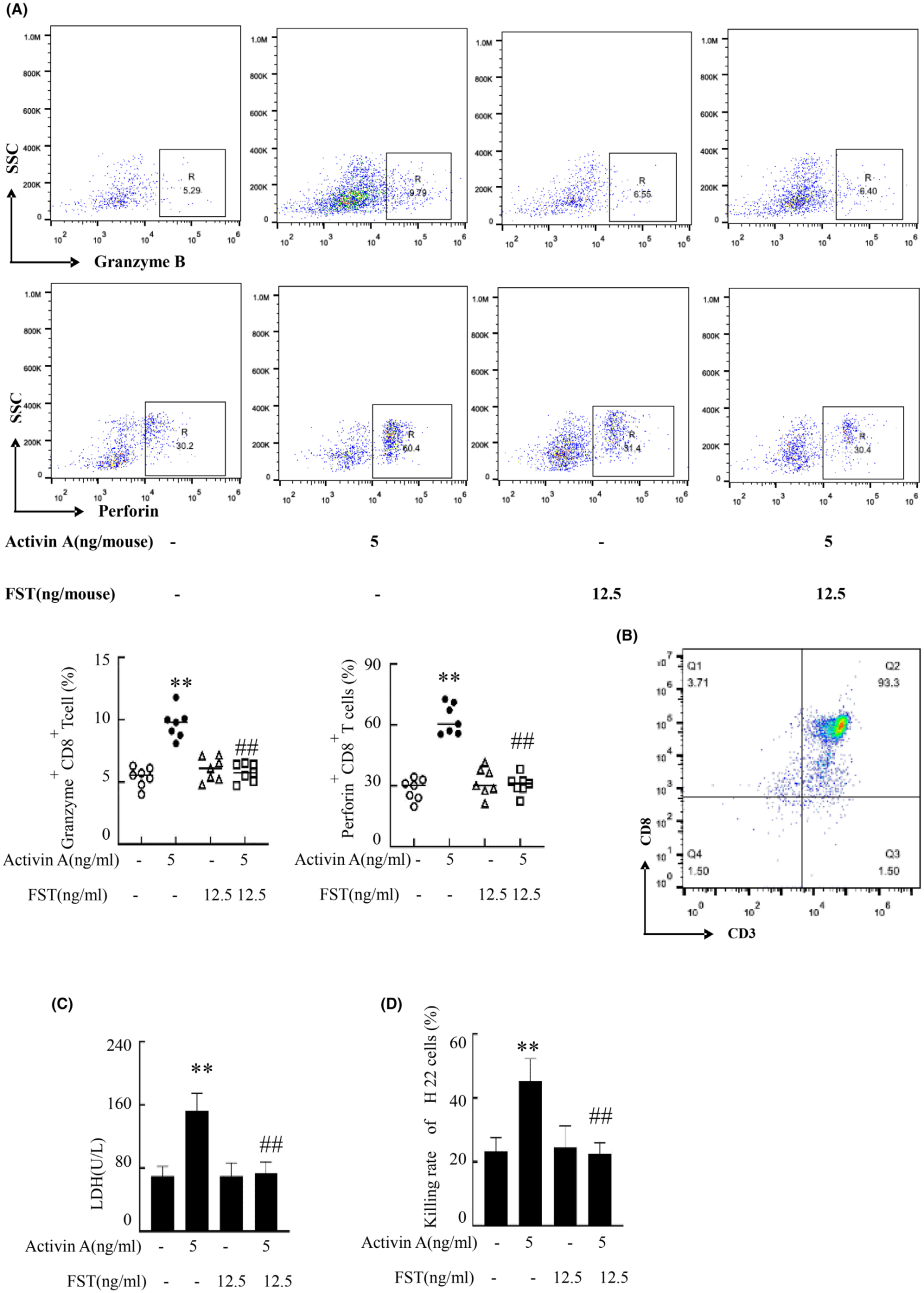
The effect of activin A on cytotoxicity of CD8^+^ T cells. (A) Tumor tissues were digested and the expression of cytotoxic particles in CD8^+^ T cells was analyzed by intracellular staining. (B)The percentage of CD3 and CD8 double positive cells in enriched cells was identified by anti‐CD3 and anti‐CD8 antibody staining. (C) The mouse tumor CD8^+^ T cells were treated with CD3/CD28 (eBioscience) followed by activin A and/or FST according to the instructions for 24 h. These cells were subsequently cocultured with H22 cells for an additional 24 h. Cytotoxicity measured through LDH release in the culture supernatant of CD8^+^ T cells. (D) The killing rate of H22 cells by CD8^+^ T cells in the presence of activin A and/or FST, respectively. The graph represented the mean ± standard deviation of seven independent experiments. Data shown as mean ± standard. **p < 0.01, compared with control group; ^##^ *p* < 0.01, compared with activin A group.

CD8^+^ T lymphocytes eliminate targets via secretion of cytotoxic particles. Intracellular staining was used to detect the regulatory effect of low‐dose activin A on cytotoxic particles expression in CD8^+^ T cells. Our findings indicated an enhanced expression in these molecules post‐activin A treatment compared to controls. However, FST significantly attenuated this stimulatory effect (Figure 4A). In addition, tumor CD8^+^ T cells were further enriched by magnetic beads. Results from flow cytometry demonstrated an approximate 93.3% positivity for CD3 and CD8 cells within the enriched population (Figure 4B). And then the cytotoxicity of intratumoral CD8^+^ T cells was examined by LDH release assay and counting H22 cell numbers. Our data confirmed that activin A augmented LDH release and CD8^+^ T cell killing efficacy against H22 cells, but FST suppressed this action (Figure 4C,D).

### 2.5 Activin A's Regulation on CD8 ^+^T cell functional markers

**FIGURE 5:**
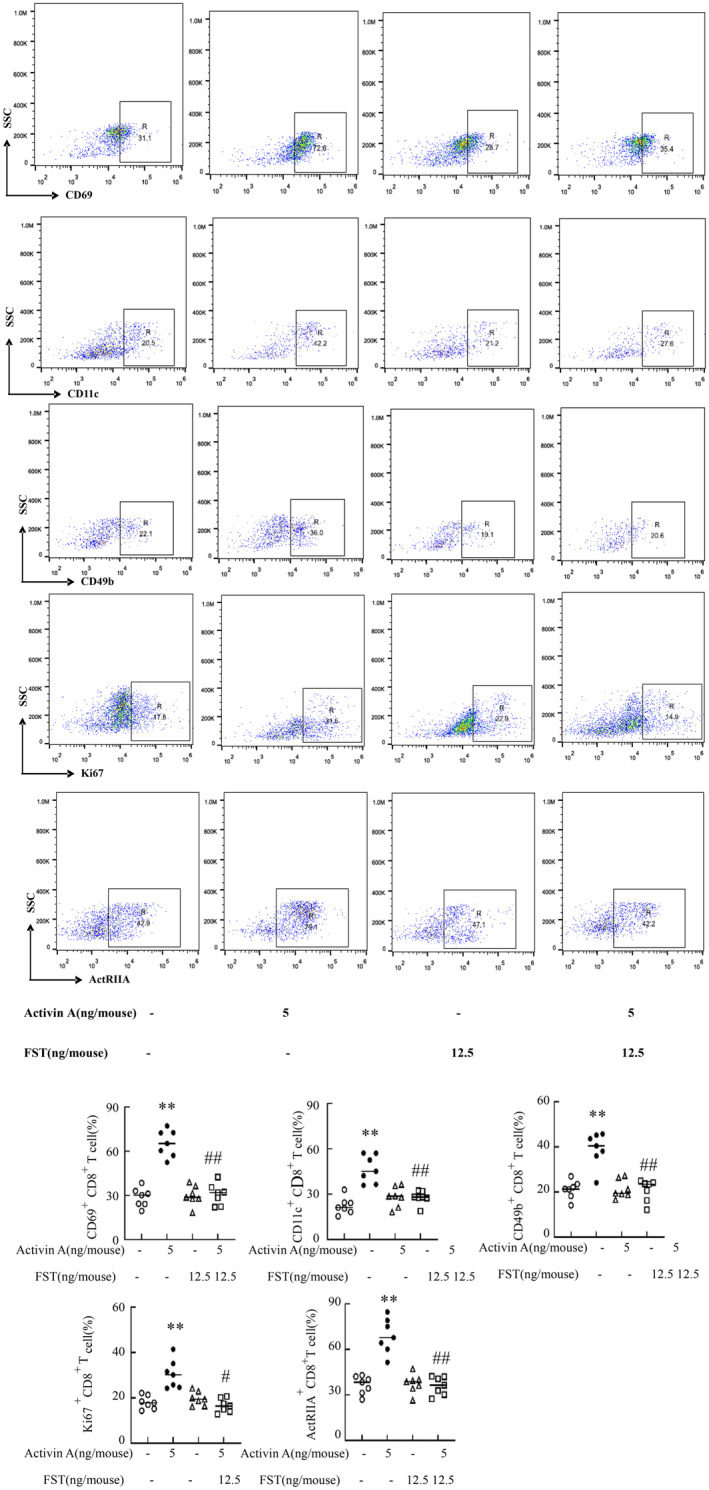
Activin A’s influence on functional molecules expression in CD8^+^ T cells. The tumor tissues lysed for flow cytometry assessment of CD69, CD11c, Ki67, CD49b, and ActRIIA in CD8^+^T cells. Data shown as mean ± standard. *p < 0.05, **p < 0.01, ***p < 0.001, compared with control group; ^#^ *p* < 0.05,^##^ *p* < 0.01, ^###^ *p* < 0.001 compared with activin A group.

To elucidate the modulatory impact of low‐dose activin A on the functional status of CD8^+^ T cells, we examined the expression of CD69, CD11c, Ki67, CD49b, and ActRIIA in CD8^+^ T cells via flow cytometry. Our findings indicated that activin A could promote the expression of CD69, CD11c, Ki67, CD49b, and ActRIIA, and the addition of FST could block activin A's promoting on these functional molecules (Figure 5).

### 2.6 Effect of activin A on the expression of ActRIIA, Smad2 and Smad3 in mouse CD8 ^+^ T cells

**FIGURE 6:**
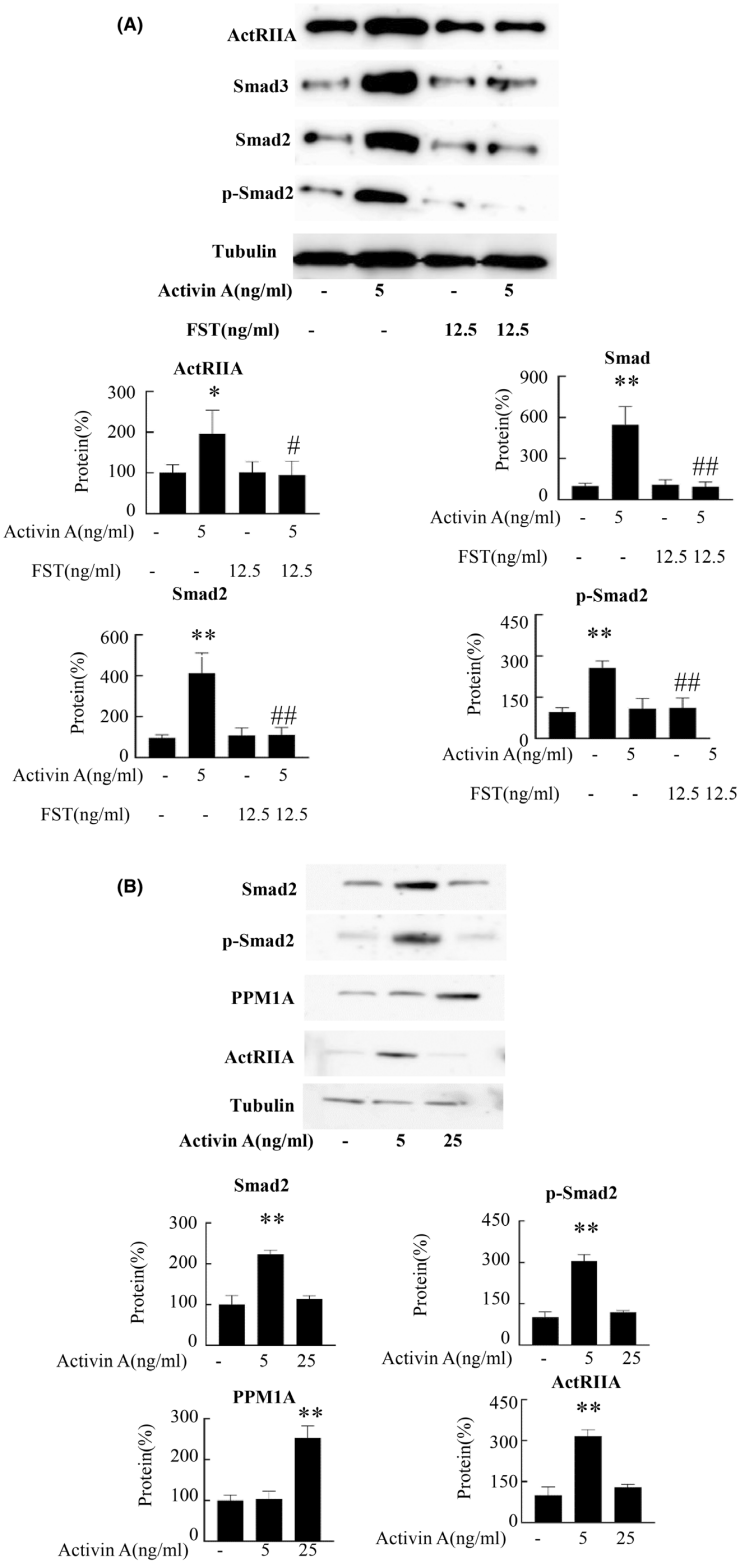
Activin A’s impact on CD8^+^T cell ActRIIA, Smad2 and Smad3 expression. (A) CD8^+^ T cells were stimulated with combinations of CD3/CD28, activin A, and (or) FST for 24 h, followed by quantification of ActRIIA, Smad3 Smad2 and p‐Smad2 protein via Western Blotting. (B) CD8^+^ T cells were treated with combinations of CD3/CD28, activin A for 24 h, and the expression of ActRIIA, Smad2, p‐Smad2 and PPM1A was deteced. Data shown as mean ± standard. *p < 0.05, **p < 0.01 compared with control group; ^#^ *p* < 0.05, ^##^ *p* < 0.01 compared with activin A group.

Smads are important protein molecules that mediate activin signal transduction.^4^ Our findings demonstrate that intratumoral CD8^+^ T cells exposed to low‐dose activin A displayed an elevated expression of ActRIIA, Smad2, and Smad3, accompanied by an increase in p‐Smad2 levels when compared with controls (Figure 6A). However, high‐dose‐activin A did not affect the expression of ActRIIA, Smad2 and p‐Smad2. But promoted the expression of PPM1A, which is a phosphatase of Smad2 (Figure 6B).

### 2.7 Increased frequency of ActRIIA ^high^ CD8 ^+^ T cells in mouse tumors

**FIGURE 7:**
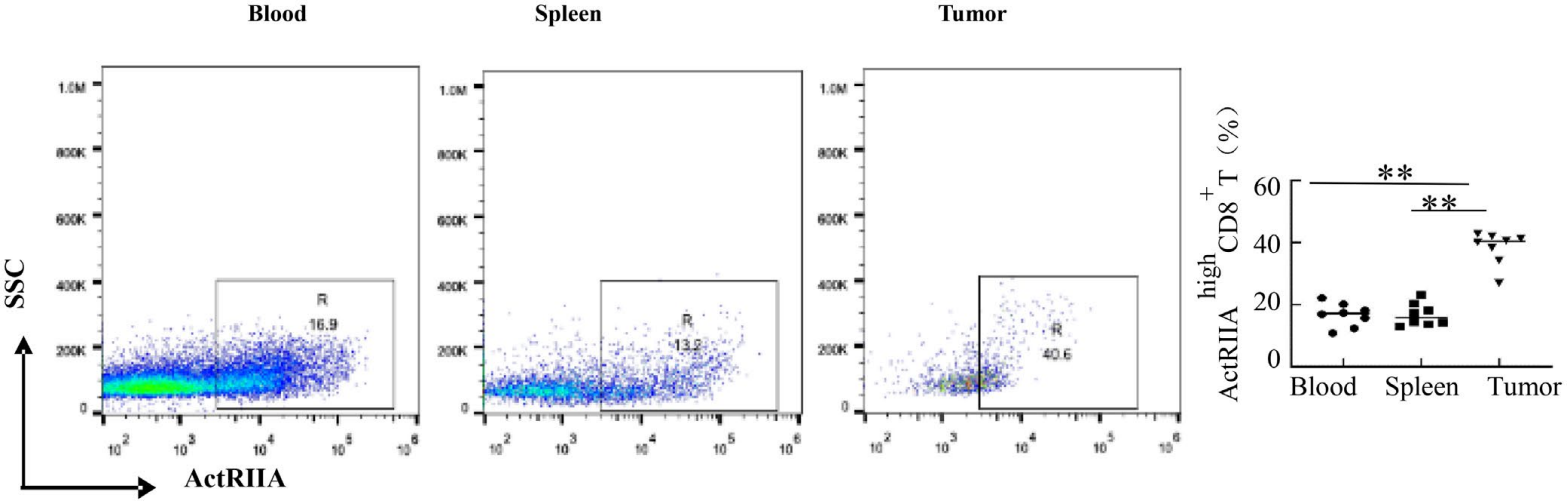
Measurement of ActRIIA on mouse CD8^+^ T cells. The level of ActRIIA on CD8^+^ T cells was detected by flow cytometry. Data shown as mean ± standard. **p < 0.01, compared with blood and spleen.

CD8^+^ T cells exhibit heterogeneity, necessitating confirmation of those bearing activin receptors. Previous results demonstrated varying ActRIIA expression in mouse CD8^+^ T cells (Figure 1C). Accordingly, mouse CD8^+^ T cells can be subdivided into ActRIIA ^high^ CD8^+^ T cells and ActRIIA ^low^ CD8^+^ T cells. We then compared the percentage of ActRIIA ^high^ CD8^+^ T among CD8^+^ T cells in peripheral blood, spleen, and tumors. The frequency of ActRIIA ^high^ CD8^+^ T cells in tumors was higher than that in peripheral blood and spleen (Figure 7).

### 2.8 Elevated cytotoxicity of ActRIIA ^high^ CD8 ^+^ T cells

**FIGURE 8:**
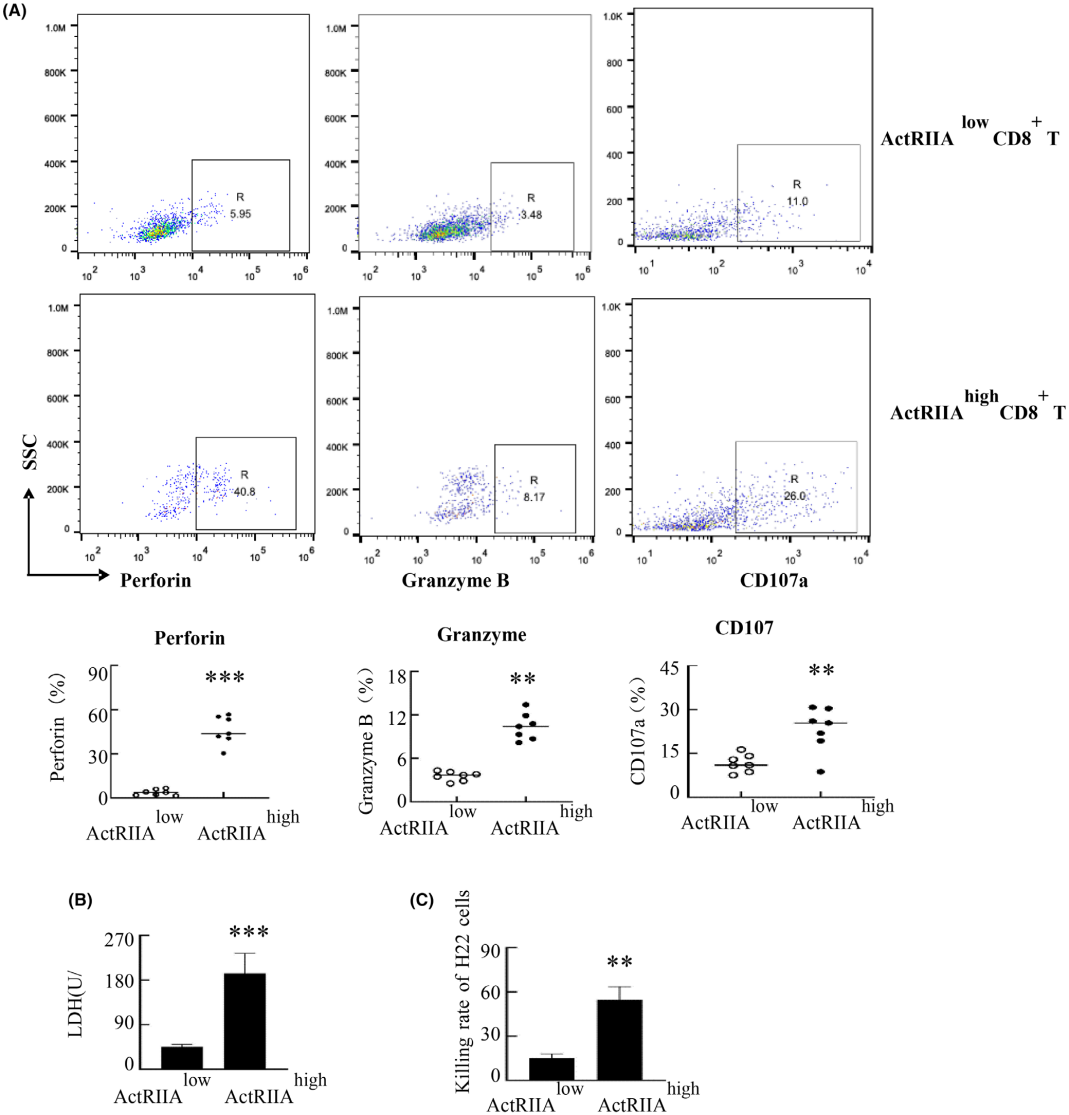
Assessment of cytotoxic potential of ActRIIA ^high^ CD8^+^ T cells. (A) Perforin, granzyme B and CD107a in ActRIIA ^high^ CD8^+^ T cells and ActRIIA ^low^ CD8^+^ T cells were analyzed via flow cytometry. (B) ActRIIA ^high^ CD8^+^ T cells and ActRIIA ^low^ CD8^+^ T cells sorted by flow cytometry were treated with CD3/CD28 for 24 h. These cells were subsequently cocultured with H22 cells for an additional 24 h. Cytotoxicity measured through LDH release in the culture supernatant of H22 cells. (C) The killing rate of H22 cells by ActRIIA ^high^ CD8^+^ T cells and ActRIIA ^low^ CD8^+^ T cells respectively. Data shown as mean ± standard. **p < 0.01, ***p < 0.001 compared with ActRIIA ^low^ CD8^+^ T cells.

To clarify the differences in cytotoxic molecule expression between ActRIIA ^high^ CD8^+^ T cells and ActRIIA ^low^ CD8^+^ T cells. Perforin, granzyme B and CD107a in ActRIIA ^high^ CD8^+^ T cells and ActRIIA ^low^ CD8^+^ T cells were profiled via flow cytometry. Observations demonstrated heightened expressions of perforin, granzyme B, and CD107a in ActRIIA ^high^ CD8^+^ T cells versus ActRIIA ^low^ CD8^+^ T cells (Figure 8A). And then the cytotoxicity of ActRIIA ^high^ CD8^+^ T cells and ActRIIA ^low^ CD8^+^ T cells was further examined by LDH release assay and counting H22 cell numbers. Research has shown that ActRIIA ^high^ CD8^+^ T cells have greater cytotoxicity than ActRIIA ^low^ CD8^+^ T cells (Figure 8B,C).

### 2.9 Enhanced cytokine‐producing capacity of ActRIIA ^high^ CD8 ^+^ T cells

**FIGURE 9:**
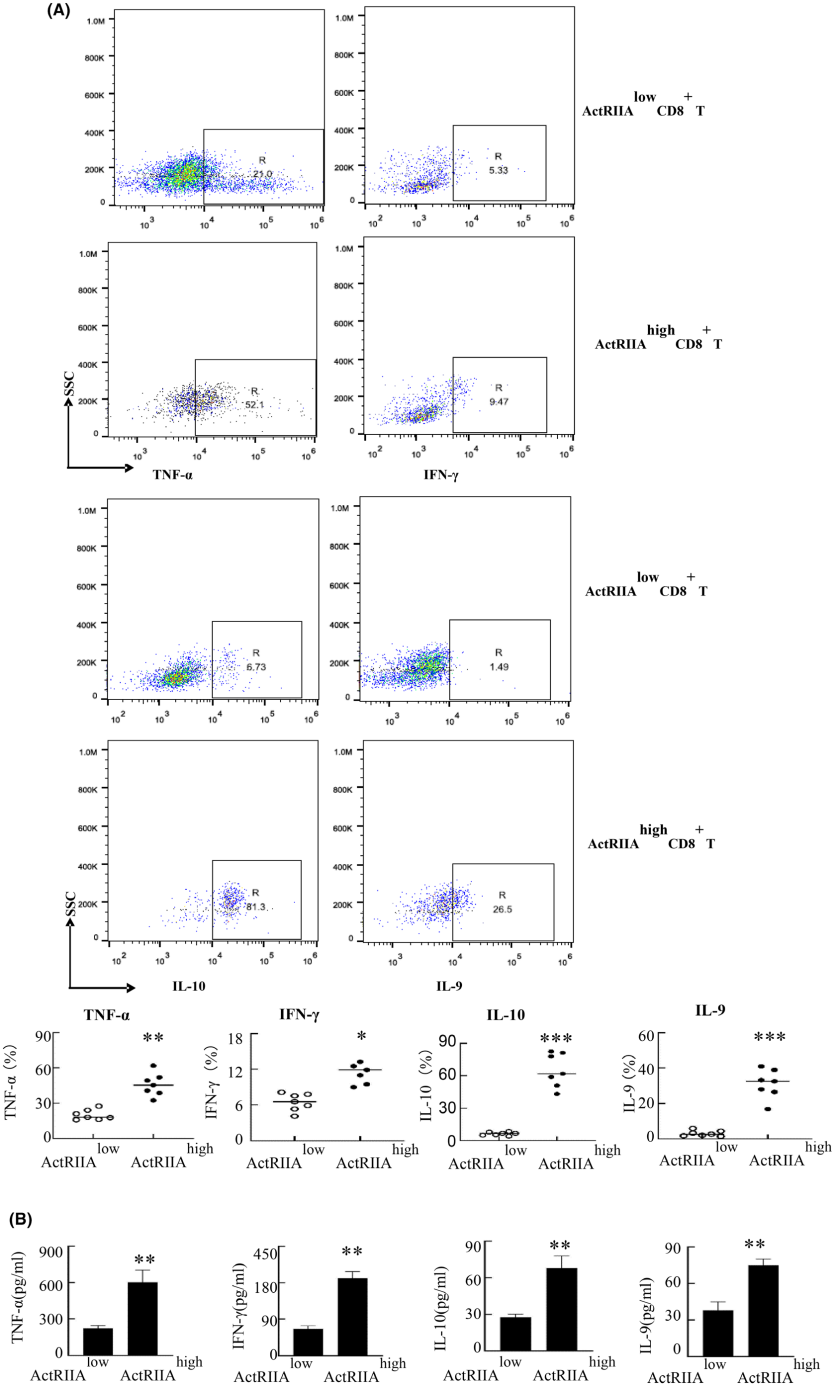
Assessment of cytokine expression in ActRIIA ^high^ CD8^+^ T cells. (A) TNF‐α, IFN‐γ, IL‐10, and IL‐9 expression in both subsets were measured via intracellular cytokine staining. (B) ActRIIA ^high^ CD8^+^ T cells and ActRIIA ^low^ CD8^+^ T cells sorted by flow cytometry were treated with CD3/CD28 for 24 h. And the content of TNF‐α, IFN‐γ, IL‐10, and IL‐9 in the culture supernatant was detected by ELISA. Data shown as mean ± standard. *p < 0.05, **p < 0.01, ***p < 0.001 compared with ActRIIA ^low^ CD8^+^ T cells.

Next, the difference in cytokine expression between ActRIIA ^high^ CD8^+^ T cells and ActRIIA ^low^ CD8^+^ T cells was analyzed via intracellular staining and ELISA. Specifically, elevated expression of TNF‐α, IFN‐γ, IL‐9, and IL‐10 were noted in ActRIIA ^high^ CD8^+^ T cells as compared to ActRIIA ^low^ CD8^+^ T cells (Figure 9A,B).

### 2.10 Phenotypic characterization of ActRIIA ^high^ CD8 ^+^ T cells

**FIGURE 10:**
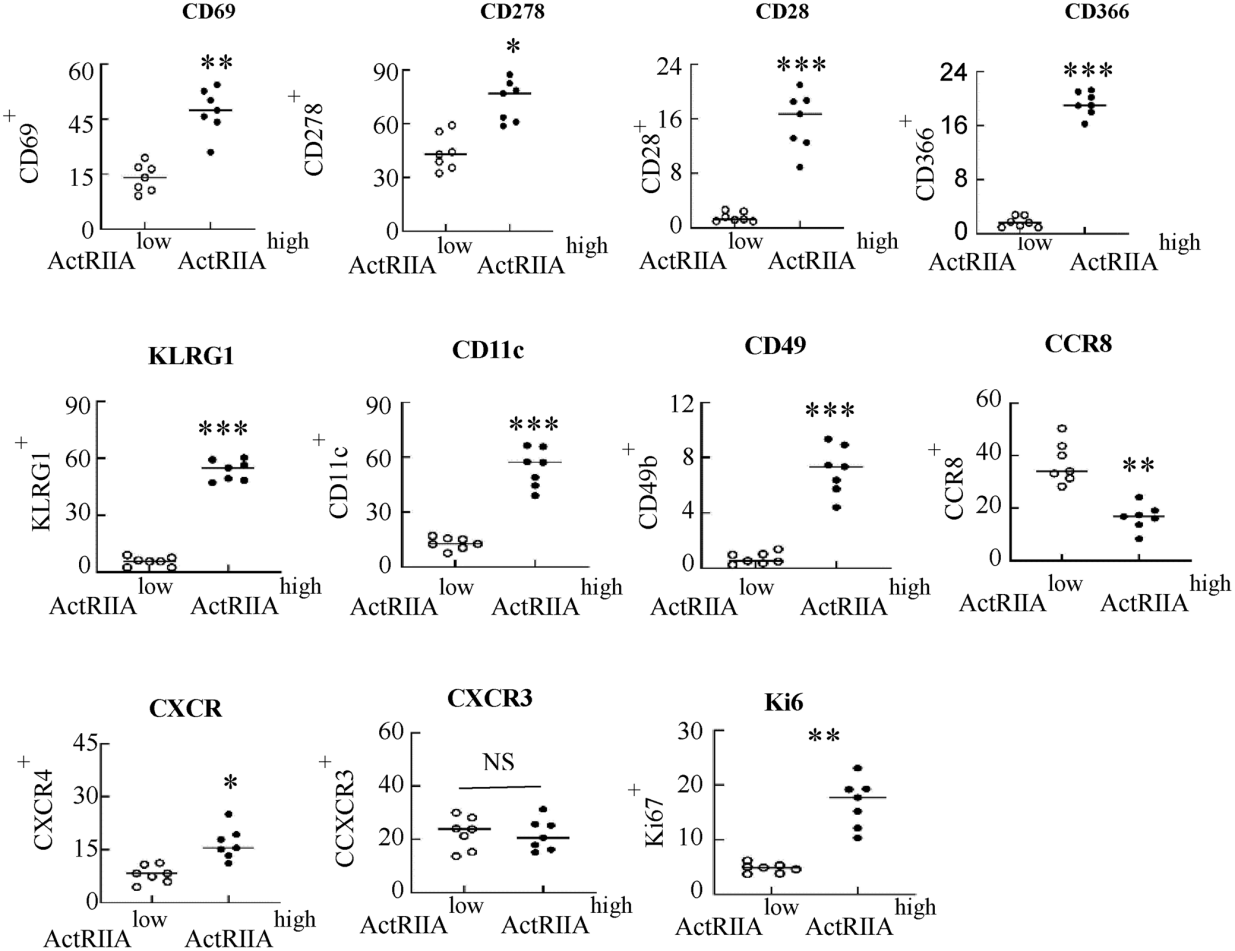
Expression of molecules that characterize the phenotype of ActRIIA^high^ CD8^+^ T cells. (A) Flow cytometry revealed distinct phenotypes for ActRIIA^low^ CD8^+^ T cells and ActRIIA^high^ CD8^+^ T cells. T Data shown as mean ± standard. *p < 0.05, **p < 0.01, ***p < 0.001, compared with ActRIIA^low^ CD8^+^ T cells; NS, not significant.

Utilizing flow cytometry, we distinguished functional surface molecules of two CD8^+^ T cell subpopulations. Firstly, ActRIIA ^high^ CD8^+^ T cells exhibit elevated expression of activation or costimulatory molecules including CD69, CD28, CD278 along with suppressing molecules inhibitory molecules CD366 and KLRG1 (Figure 10). Secondly, ActRIIA ^high^ CD8^+^ T cells express a set of cell adhesion and tissue localization indicators such as CD69, CD11C, and CD49b (Figure 10). Thirdly, CXCR4 was highly expressed in ActRIIA ^high^ CD8^+^ T cells, while CCR8 expression was downregulated, and no difference in CXCR3 expression (Figure 10). Finally, ActRIIA ^high^ CD8^+^ T cells upregulated Ki67 expression (Figure 10). Representative scatter plot is shown in the Figure S2. We are using bioinformatics methods to analyze TCGA—LIHC data found ActRIIA closely related with the expression of tumor immune molecules, detailed data is shown in the Figure S3.

### 2.11 Mettle3 inhibits the expression of ActRIIA in CD8 ^+^T cells

**FIGURE 11:**
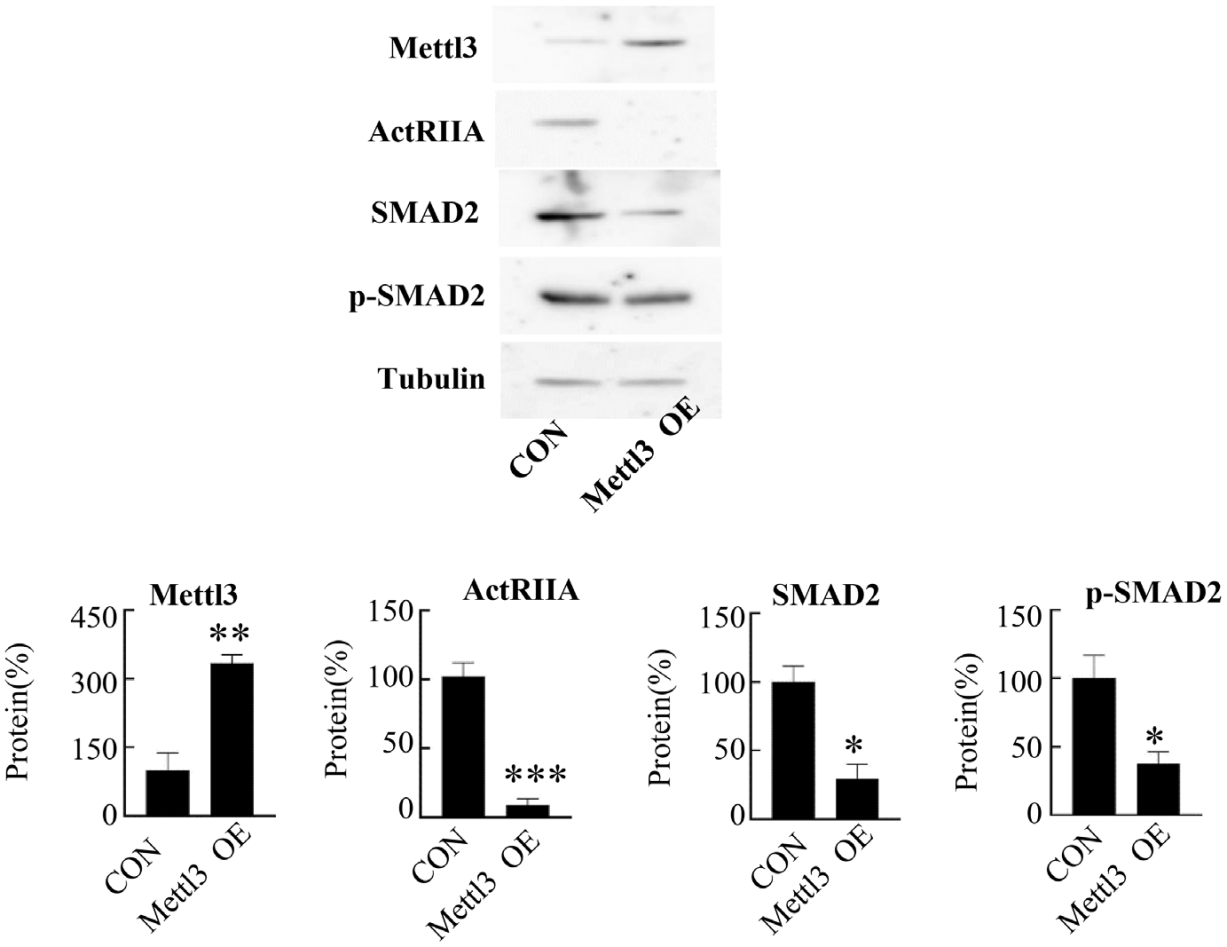
Overexpressed Mettl3 affect the protein expression in intratumoral CD8^+^T cells. The intratumoral CD8^+^T cells of mouse were treated with CD3/CD28, Lentivirus‐mediated overexpressed Mettl3 for 72 h, and then the levels of ActRIIA, Smad2 and p‐Smad2 proteins were examined by Western blotting. *p < 0.05, **p < 0.01, ***p < 0.001 comparing to control.

Previous report has shown that Mettl3 can inhibit the expression of ActRIIA in cells.^40^ We found that overexpression of Mettl3 inhibited the expression of ActRIIA in CD8^+^ T cells. Moreover, overexpression of Mettl3 results in decreased expression of p‐Smad2 and Smad2 in CD8^+^ T cells (Figure 11).

## 3 DISCUSSION

Activin A has a wide range of functions. In addition to its role in biological processes such as hematopoiesis and development process of embryos, it also impacts microbial host defense, allergic diseases, autoimmune diseases and cancer.^6^, ^15^, ^16^ Immune cells, such as dendritic cells, lymphocytes, macrophages, and NK cells, express activin receptors and downstream related signaling molecules, which belong to activin A responsive cells.^17^, ^18^, ^19^, ^20^ Our research revealed that ActRIIA, ActRIIB, Smad2, 3,4 mRNA are discernible in CD8^+^ T cells. Meanwhile, CD8^+^ T cells in different organs and tissues expressed ActRIIA receptor to varying degrees. In addition, activin A regulated ActRIIA, Smad2 and Smad3 and p‐Smad2 expression in CD8^+^ T cells, which further verified that mouse CD8^+^ T cells were also activin A responsive cells.

Activin A levels are high in some physiological and pathological states, so many studies have applied 30, 50 ng/mL or even higher dose of activin A to the research process of related diseases.^11^, ^21^ However, the level of activin A is low in various biological processes of the body.^12^ Hence, we opted for low‐dose activin A akin to these natural conditions. Our findings confirmed high‐dose activin A stimulates tumor progression and attenuates CD8^+^ T cell presence, echoing prior reports,^11^ while low‐dose activin A had the opposite effect. Liver cancer patients with low serum levels of activin A have a longer survival period, and activin A has the potential to become a biological marker for predicting the recovery of liver cancer patients. Furthermore, activin A augmented CD4^+^ T cell abundance within the tumor but had no influence on NK cell count. CD8^+^ T cell depletion negated the impact of activin A. H22 does not express ActRIIA, rendering activin A ineffective on its proliferation and apoptosis of H22. Activin A may affect tumor growth by regulating CD8^+^ T cells.

Endogenous FST is a binding protein capable of counteracting the paracrine effects of activin.^22^, ^23^ By sequestering activin, FST obstructs its interaction with the cellular activin receptors, preventing the associated biological outcomes.^23^, ^24^ FST inhibitted the effect of activin A on tumor growth, further indicating that low‐dose activin A inhibited tumor growth. According to the above results, low‐dose activin A may play an active immunological function when the body is attacked by pathogens or tumor invasion.

CD8^+^ T cells can lyse target cells and inhibit tumor growth by secreting cytotoxic granules such as perforin and granzyme B.^25^ Activin A promoted the expression of cytotoxic components including perforin and granzyme B in CD8^+^ T cells. In vitro experiments indicated that this cytokine augments CD8^+^ T cell cytotoxicity against H22 cells, demonstrating its potential to enhance their elimination potency. In addition, low‐dose of activin A increased CD69 expression, a hallmark of Trm,^26^, ^27^ and also promoted CD11c and CD49b expression, suggesting that low‐dose activin A may promote CD8^+^ T cell tissue colonization function. It is well known that Ki67 is a marker of cell proliferation.^28^ And low‐dose activin A upregulated Ki67 expression in CD8^+^ T cells, suggesting it may elevate the tumoral CD8^+^ T cell count by stimulating their proliferation. Consistently, previous studies demonstrated activin A as a potent regulator for the expression of ActRIIA in NK cells,^29^ with our findings corroborating this observation within CD8^+^ T cells. Activin A likely exerts a chemotactic effect on CD8^+^ T cell migration to tumors, or can enhance the expression of ActRIIA in these cells, necessitating further investigation. PPM1A as a phosphatase, can reduce the protein level of p‐smad2 in cells, thereby affecting the TGF signaling process.^30^ Our found that low‐dose activin A promoted the expression of p‐Smad2, and Smad2 in CD8^+^ T cells, but has no effect on PPM1A. However, high‐dose‐activin A did not affect the expression of Smad2 and p‐Smad2, but promoted the expression of PPM1A. This may be the mechanism by which low‐dose A and high‐dose A have different regulatory effects on CD8^+^ T cells.

Immune cells are highly heterogeneous, and human NK cells can be distinguished into CD56^bright^ and CD56^dim^ NK cells by CD56, which have been identified and well characterized.^31^, ^32^ Notably, human CD8^+^ T cells are classified into subpopulations via PD1, and PD1^Hi^CD8^+^ T cells have a great relationship with the prognosis of hepatocellular carcinoma.^13^ Certain CD8^+^ T cells expressing CD11c exhibit superior efficacy for HIV‐1 control in immunotherapy.^33^ Based on the fact that CD8^+^ T is a heterogeneous immune cell population, CD8^+^ T cells in mice could be divided into two distinct cell populations by ActRIIA. The number of ActRIIA ^high^ CD8^+^ T cells in tumors surpasses those in peripheral blood and spleen, indicating their potential origin from these sites.

CD8^+^ T cell cytotoxic molecules, cytokines, and receptors demonstrate variable expression during tumorigenesis.^34^, ^35^ ActRIIA ^high^ CD8^+^ T A cells exhibit potent cytotoxicity against H22 cells, concurrently demonstrating an exceptional cytokine production capacity. Additionally, ActRIIA ^high^ CD8^+^ T cells express elevated levels of activation or costimulatory molecules CD69, CD278, CD28, and CD49b, along with inhibitory receptors CD366 and KLRG1. ActRIIA ^high^ CD8^+^T cells display activation, providing protection against the tumor's destructive effects whilst sparing the host. These cells were elevated within the tumor. We further explored the mechanism of this phenomenon. ActRIIA ^high^ CD8^+^ T cells expressed CD69 CD11c CD49b, indicative of their tissue resident feature in the tumor. We also observed an increase in the expression of chemokine receptors CXCR4 and CCR7, but no change in CXCR3. Research indicates that tumor tissues expressed ligands for these receptors of chemokines, with the interactions of CXCL12‐CXCR4, CXCL10‐CXCR3, and CCL19 –CCR7 play crucial role in attracting CD8^+^ T cells to infiltrate tumors.^36^, ^37^, ^38^, ^39^ Variations in the expression of chemokine receptors may be the cause of the uneven distribution of ActRIIA ^high^ CD8^+^ T cells in vivo. In addition, ActRIIA ^high^ CD8^+^ T expresses Ki67 highly, and this cell population may have a high proliferative potential. In conclusion, the enrichment of ActRIIA ^high^ CD8^+^ T cells in tumors is likely the result of a complex interplay between multiple factors, and these cells appear to contribute positively to anti‐tumor effects. Study has reported that METTL3 could repress ActRIIA synthesis.^40^ Overexpression of METTL3 in CD8^+^ T cells inhibits the expression of ActRIIA, which may be the mechanism leading to differential expression of ActRIIA by CD8^+^T cells.

These results demonstrate that varying activin A levels regulate CD8^+^ T cell activity within tumors, impacting tumor outcomes. Within a range, low‐dose activin A promotes CD8^+^ T cell anti‐tumor potency; however, exceeding a specific threshold results in an opposing effect. This suggests that the level of activin A could potentially serve as a predictive indicator for tumor progression and warrants further exploration. Additionally, this study identified ActRIIA ^high^ CD8^+^ T cells possessing potent antitumor activity and express multiple activation and inhibitory receptors. Research indicates blocking immunosuppressive receptor expression enhances T cell anti‐tumor function. However, the clinical response rate is relatively low, for example, only 15%–20% of HCC patients respond to PD1 blockade.^41^ Consequently, further investigation into combining ActRIIA ^high^ CD8^+^ T cells, immune suppressor blockers, and costimulatory molecular stimulators for cancer therapy is necessary.

## 4 CONCLUSION

Our study indicated that low‐dose of activin A augmented the content of CD8^+^T cells within the tumor and promote cytotoxicity of CD8^+^T cells, thereby suppressing tumor growth. Furthermore, our research established that ActRIIA ^high^ CD8 ^+^ T cells actively contribute to tumor immunity.

## 5 LIMITATIONS OF THE STUDY

In this investigation, exogenous activin A administration elevated the ActRIIA ^high^ CD8^+^ T cells content in hepatoma, yet various immune cells produce endogenous activin A. Limitations include the neutralization of endogenous activin A, impeding the differentiation of autocrine versus paracrine activin A influence on CD8^+^ T cell responses. Evidence suggests that activin A levels are correlated with hepatocellular carcinoma progression. However, the role of ActRIIA ^high^ CD8^+^ T cells as a prognostic indicator, reflecting hepatoma severity, remains elusive, necessitating further clinical research.

## 6 EXPERIMENTAL MODEL DETAILS

### 6.1 Mice

Male C57BL/6 mice from 8 to 10 weeks were provided by the animal center of Weifang Medical University, China. All animal experiments were conducted in accordance with the Institutional Animal Care and Use Committee of Weifang Medical University, and all experimental protocols were approved by the Institutional and Licensing Committee of Weifang Medical University (2023SDL080).

### 6.2 Cancer cell lines and cell culture

H22 mouse hepatoma cell line was provided by Weifang Medical University, China. Cells were cultured in an RPIM 1640 culture medium (Gibco, Carlsbad, CA) and supplemented with 10% (V/V) fetal bovine serum (FBS, Invitrogen, Carlsbad, CA) at 37°C with 5% CO_2_.

## 7 METHOD DETAILS

### 7.1 Isolation of the CD8 ^+^ T cells

Mouse tumors were dissociated by Collagenase (1 mg/mL) and DNAse I (0.2 mg/mL), and spleen were dissociated with 70 μm cell strainer to make into single cell suspension. And then the single cell suspension was centrifuged with 70% Percoll at 1200 rpm for 20 min to isolate mononuclear cells. After collecting the middle cell layer and washing, mouse spleen CD8^+^ T cells were separated by magnetic bead sorting kit according to the instructions. The percentage of CD3 and CD8 positive cells in the enriched cells was identified by flow cytometry.

### 7.2 Measurement of apoptosis

H22 cells were seeded in a 24‐well plate with a density of 5 × 10^4^/mL in each well, saline, activin A 1 ng/mL, activin A 5 ng/mL, and activin A 25 ng/mL were added and incubated for 24 and 48 h in 5% CO_2_ at 37°C. Cells were harvested and stained with Annexin V‐FITC/7‐AAD (Procell, Wuhan, China) as the manufacturers' instructions. The percentages of dead cells were measured by flow cytometry.

### 7.3 Cell counting kit‐8 (CCK‐8) assay

H22 cells were seeded in a 96‐well plate with a density of 2 × 10^4^ /mL in each well, saline, activin A 1 ng/mL, activin A 5 ng/mL, and activin A 25 ng/mL were added and incubated for 24 and 48 h in 5% CO_2_ at 37°C, and the cell proliferation was detected according to the instructions of Cell Counting Kit‐8 (Beyotime, Shanghai, China).

### 7.4 Analysis of CD8 ^+^ T cells cytotoxicity

CD8^+^ T cell cytotoxicity was evaluated by the lactate dehydrogenase (LDH) release method and the amount of H22 cells was counted. The CD8^+^ T cells of tumor (1 × 10^5^/well) were plated in 96‐well plate, and incubated with CD3/CD28 (eBioscience) followed by activin A and/or FST according to the instructions for 24 h. Then the CD8^+^ T cells (1 × 10^5^ cells/well) were cocultured with mouse tumor cell H22(1 × 10^5^ cells/well) for 24 h. The LDH released into the media from damaged cells was measured using LDH cytotoxicity assay kit (Nanjing Jiancheng Bioengineering Institute, China). In order to further confirm the CD8^+^ T cell‐dependent cytotoxicity, the cells were collected and the amount of H22 cells was counted. The killing rate of H22 cells by CD8^+^ T cells was calculated by the following equation. Killing rate = (total amount of H22 cells in control group without CD8^+^ T cells—the amount of H22 cells in experiment group with CD8^+^ T cells/total amount of H22 cells in control group without CD8^+^ T cells) × 100%.

### 7.5 Tumor growth and treatment

C57BL/6 J mice were inoculated with 1 × 10^6^ H22 tumor cells on the right flank on Day 0. From Day 7, an equal volume of saline, activin A, and/or FST was injected directly into the tumor daily. Tumor volumes were measured with a caliper by the length (L) and width (W), and calculated as tumor volume = (L × W^2^)/2. Animals were considered dead when tumor volume reached >1500 mm^3^.

### 7.6 Flow cytometry analysis

Mouse tumors were dissociated by Collagenase (1 mg/mL) and DNAse I (0.2 mg/mL), and lymph nodes and spleen were dissociated with 70 μm cell strainer. Anti‐Fc‐γ III/II was added and incubated at room temperature for 20 min to block the Fc receptor, followed by staining with selective antibodies of cell surface markers. Intracellular markers including perforin, granzyme B, and Ki67 were stained after cell permeabilization with Cyto‐Fast™Fix/Perm Buffer Set (BioLegend). Data were collected on BD FACSCalibur flow cytometer and analyzed by FlowJo (Tree Star Inc., Ashland, OR) software.

### 7.7 CD107a detection assay

Tumors from mice were isolated under sterile conditions, digested to obtain single cell suspensions, and then examined for intracellular CD107a expression in CD8^+^ T cells as previously reported^13^.

### 7.8 Intracellular cytokine staining

The single cell suspension obtained after tumor digestion was cultured for 5 h with eBioscience™ Cell Stimulation Cocktail (plus protein transport inhibitors). The collected cells were washed twice and stained with anti‐CD45 antibody, anti‐CD3 antibody, anti‐CD8 antibody, and anti‐ActRIIA antibody for 30 min. Foxp3 staining buffer set (eBioscience) was added and incubated at room temperature for 20 min, followed by IL‐10, TNF‐α, IFN‐γ, and IL‐9 staining for 30 min. Data were collected on BD FACSAria™III flow cytometer and analyzed by FlowJo (Tree Star Inc., Ashland, OR) software.

### 7.9 ELISA

Peripheral blood was collected from mice, and blood samples were transferred to EDTA‐containing blood collection vessels and centrifuged to obtain peripheral blood plasma. The plasma activin A and FST content was detected according to the operating instructions of the ELISA detection kit.

### 7.10 Western blotting

Mouse CD8^+^ T cells (5 × 10^6^) were cultured with CD3/CD28, activin A and/or FST for 48 h. Cell lysate was added to the collected cells, and the supernatant was taken after centrifugation. The proteins in the supernatant were separated by SDS‐PAGE and transferred onto a polyvinylidene difluoride membrane (PVDF), rabbit anti‐ActRIIA antibody (1:1000), rabbit anti‐Smad3 antibody (1:500), rabbit anti‐Smad2 antibody (1:500), rabbit anti‐phosphorylated Smad3 (p‐Smad2) antibody (1:500) and the rabbit anti‐Tubulin antibody (1:1000) were added and incubated for 2 h. After washing 3 times, horseradish peroxidase‐ labeled goat anti‐rabbit IgG was added for 1 h, washed 3 times, and finally, ECL (ECLPlus; Amersham Pharmacia Biotech) reagent was added to detect the target protein by chemiluminescence imaging.

### 7.11 qRT‐PCR

RNA was extracted from purified mouse spleen CD8^+^ T cells. Total RNA was reverse transcribed into cDNA according to the instructions of the FastKing One‐step Genome Removal kit (Tiangen, Beijing, China). The qRT‐PCR experiment was performed according to the instructions of the SuperReal Fluorescent Quantitative Premix Reagent Enhanced Kit (Tiangen, Beijing, China). The primer sequences are provided in Table 1.

**TABLE 1:** Primers sequences were used in qRT‐PCR.

Target gene	Forward sequence (5′→3′)	Reverse sequence (5′→3′)
*Acvr2a*	GGTGATAAAGATAAACGGCGAC	AGTTGATATCATCCAGCCAACA
*Acvr2b*	CTTCCTGCGTATCGACATGTA	AGCATGTACTCATCGACAGG
*Smad2*	CTCTCCAACGTTAACCGAAATG	CACCTATGTAATACAAGCGCAC
*Smad3*	ATTCCATTCCCGAGAACACTAA	TAGGTCCAAGTTATTGTGTGCT
*Smad4*	AGTTCACAATGAGCTTGCATTC	TTCAAAGTAAGCAATGGAGCAC
*GAPDH*	GGCAAATTCAACGGCACAGTCAAG	TCGCTCCTGGAAGATGGTGATGG

### 7.12 Quantification and statistical analysis

All data are expressed as means ± SD. Statistical comparisons were performed using oneway analysis of variance, followed by Scheffe's test using SPSS 19.0 software (IBM Corp., Armonk, NY, USA), and *p* < 0.05 was considered statistically significant.

## 8 KEY RESOURCES TABLE

**Table:** 

Antibodies	Source	Identifier
Mouse monoclonal Percp‐Cy5.5 conjugated anti‐CD3	BioLegend	CAT#100217
Mouse monoclonal PE conjugated anti‐CD8a	STEMCELL	CAT#60023PE.1
Mouse monoclonal PE conjugated anti‐CD4	Elabscience	CAT#E‐AB‐F1097D
Mouse monoclonal Percp‐Cy5.5 conjugated anti‐NK1.1	BioLegend	CAT#108727
Mouse monoclonal FITC conjugated anti‐Geanzyme B	BioLegend	CAT#515403
Mouse monoclonal FITC conjugated anti‐Perforin	Elabscience	CAT#E‐AB‐F1294C
Mouse monoclonal FITC conjugated anti‐CD69	BioLegend	CAT#104505
Mouse monoclonal FITC conjugated anti‐CD11c	Elabscience	CAT#E‐AB‐F0991C
Mouse monoclonal FITC conjugated anti‐CD49b	BioLegend	CAT#108906
Mouse monoclonal Percp‐Cy5.5 conjugated anti‐CD107a	BioLegend	CAT#121625
Mouse monoclonal PE conjugated anti‐TNF‐α	BioLegend	CAT#506305
Mouse monoclonal Percp‐Cy5.5 conjugated anti‐IFN γ	Invitrogen	CAT#45–7311‐80
Mouse monoclonal FITC conjugated anti‐IL 10	Elabscience	CAT#E‐AB‐F1197C
Mouse monoclonal PE/Cyanine7 conjugated anti‐CD45	BioLegend	CAT#157206
Mouse monoclonal PE conjugated anti‐IL 9	BioLegend	CAT#100230
Mouse monoclonal FITC conjugated anti‐CD278	BioLegend	CAT#313505
Mouse monoclonal PE conjugated anti‐CD366	BioLegend	CAT#134003
Mouse monoclonal FITC conjugated anti‐KLRG1	BioLegend	CAT#138409
Mouse monoclonal PE conjugated anti‐CCR8	BioLegend	CAT#150311
Mouse monoclonal PE conjugated anti‐CXCR3	BioLegend	CAT#155903
Mouse monoclonal PE conjugated anti‐CXCR4	BioLegend	CAT#153805
Mouse monoclonal FITC conjugated anti‐Ki67	BioLegend	CAT#151211
Mouse monoclonal APC conjugated anti‐hActivinRIIA	R&D	CAT#FAB340A
Mouse monoclonal FITC conjugated anti‐CD3	BioLegend	CAT#300405
Mouse monoclonal FITC conjugated anti‐CD8a	BioLegend	CAT#100706
SMAD2 Polyclonal antibody	Proteintech	CAT#12570‐1‐AP
Phospho‐SMAD2 (Ser465/Ser467) (E8F3R) Rabbit mAb	Cell Signaling Technology	CAT#18338
SMAD3 Monoclonal antibody	Proteintech	CAT# 66516
ACVR2A Rabbit Polyclonal Antibody	Bioss	CAT#bs‐22941R
Alpha Tubulin Polyclonal antibody	Proteintech	CAT#11224
*Chemicals and recombinant proteins*		
eBioscience™ Cell Stimulation Cocktail (plus protein transport inhibitors)	eBioscience	CAT#00–4975‐93
Recombinant Human/Mouse/Rat Activin A Protein	R&D	CAT#338‐AC
Recombinant Mouse Follistatin 288 (FS‐288) Protein	R&D	CAT#769‐FS
*Critical commercial assays*		
Mouse Activin‐A ELISA kit	MEIMIAN	MM‐0205 M1
Mouse FST ELISA kit	MEIMIAN	MM‐0403 M1
Cell Counting Kit‐8	Beyotime	C0038
RNApre pure Cell/Bacteria Kit	TIANGEN	DP430
Omni‐ECL™Enhanced Pico Light Chemiluminescence Kit	Epizyme	SQ101
Cyto‐FastTMFix/Perm Buffer Set	BioLegend	426,803
DYNABEADS MOUSE T‐ACT CD3/CD28	eBioscience	11456D
EasySep™ Mouse CD8a Positive Selection Kit II	STEMCELL	18,953
LDH cytotoxicity assay kit	Nanjing Jiancheng Bioengineering Institute	A020‐1

## AUTHOR CONTRIBUTIONS

**Chunhui Ma:** Data curation (equal); writing – original draft (equal); writing – review and editing (lead). **Liangchang Hu:** Formal analysis (equal). **Xuguang Zhang:** Writing – review and editing (equal). **Yu Zhao:** Methodology (equal); software (equal).

## CONFLICT OF INTEREST STATEMENT

The authors declare no conflict of interest.

## Supporting information


Data S1:

## Data Availability

The data that support the findings of this study are available on request from the corresponding author upon reasonable request.
