# Supplementary material for: Activin A promoted the anti‐tumor effect of ActRIIA high CD8 + T cells in mouse hepatoma
Source: Cancer Med. 2024 Dec 27;14(1):e70147. doi: 10.1002/cam4.70147 (PMC11672029; doi:10.1002/cam4.70147)
Supplement: Supplementary file 1 — Data S1: [file CAM4-14-e70147-s001.docx]

Supplementary materials

Supplemental figure 1：

1.1. Study population

The study includes a total of 162 patients with primary hepatocellular carcinoma who received therapy at the Department of liver, at the Affiliated Hospital of Shandong Second Medical University between 2019 and 2023. And 156 Were Followed Up Actually. The study was approved by the Affiliated Hospital of Shandong Second Medical University and complies with the principles of the Declaration of Helsinki(2024SDL174).

1.2. Collection of peripheral blood samples

Blood samples of primary hepatocellular carcinoma patients were collected prior to the admission of therapy. Serum samples were centrifuged within 2 h after collection at 1800×g for 10 min and aliquoted before storage at −80 ◦C.

1.3. ELISA for activin A levels

The serum activin A levels were determined using the activin A Human ELISA Kit (#ml026146, Mlbio, ShangHai, China). The experiment was conducted according to the manufacturer’s instructions. Samples were analyzed in triplicates.

We determined the median activin A levels of the 156 primary hepatocellular carcinoma patients at baseline prior to futher analysis. The activin A levels ranged from 300 pg/mL to 5,000 pg/mL with a median of 2,400 pg/mL in our study cohort. Stratification according to the suggested cutoff resulted in 76 HCC patients in the low-activin A group (<2400 pg/mL) and 80 in the high-activin A group (≥2400 pg/mL). The analysis using the log-rank test demonstrated that liver cancer patients with high activin A have an impaired OS with a median of 419 days (95% CI: 0.30–0.85) compared to patients with a low activin A with a median of 821 days (95% CI: 1.16–3.28) (p =0.0092) (Figure 1).

0 500 1000 1500 2000

0.0

0.5

1.0


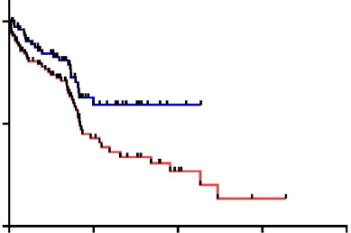


Probability of Survival


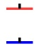


Low activin A

High activin A

Supplemental figure1. Kaplan–Meier survival curves displaying the overall survival in HCC patients (n = 156).

Univariate analysis was carried out via the log-rank test (Mantel–Cox). A p-value < 0.05 was considered statistically significant.

Supplemental figure 2:

The gating strategies for CD8^+^T cells in mouse tumors is shown. Firstly, DAPI is used to remove dead cells, and then the CD45^+^CD3^+^CD8^+^method is used to gate CD8^+^T cells.


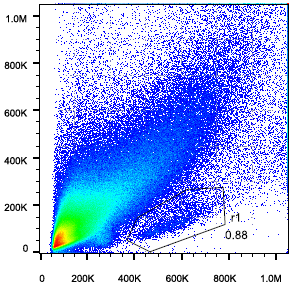


FSC

SSC

Isotype IgG

SSC


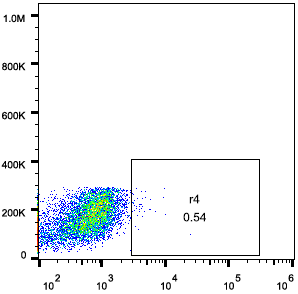


CD45

SSC


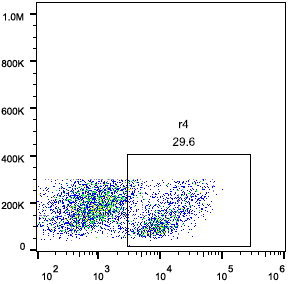


DAPI

SSC


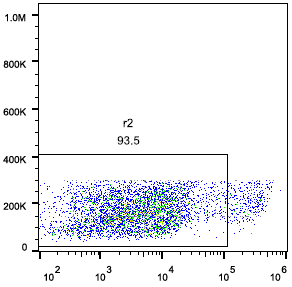


Isotype IgG

Isotype IgG


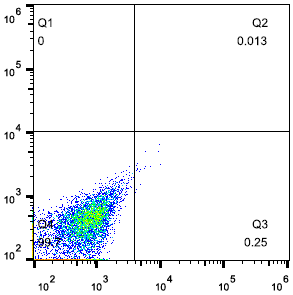


CD3

CD8


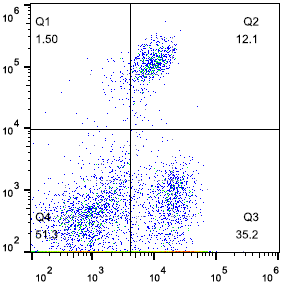


Supplemental figure 3:

The expression of ActRIIA ^high^ CD8 ^+^ T cells and ActRIIA ^low^ CD8 ^+^ T cells molecules was detected by flow cytometry, and the representative scatter plot is shown below.

**CD69 CD278 CD28**

**SSC**


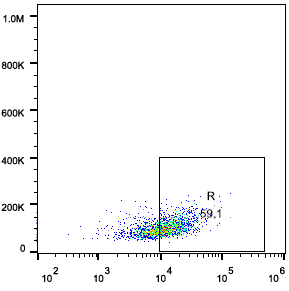

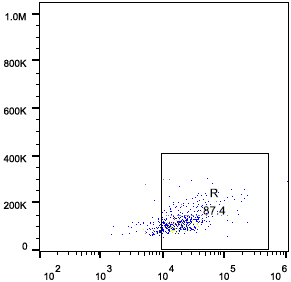

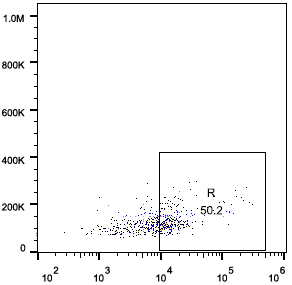

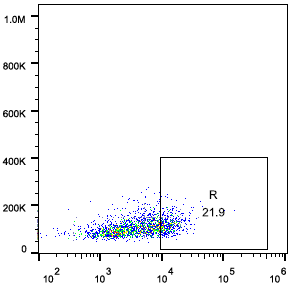

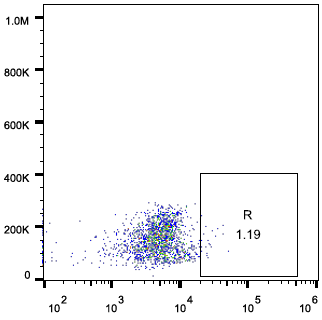

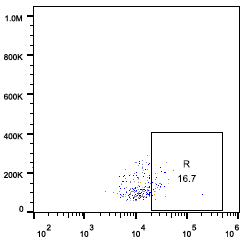


**ActRIIA^low^CD8^+^ T**

**ActRIIA^high^CD8^+^ T**

**CD366 KLRG1 CD11c**

**SSC**


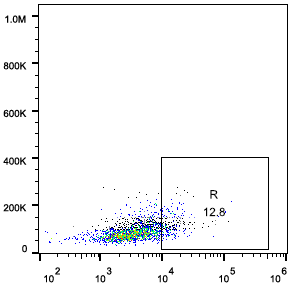

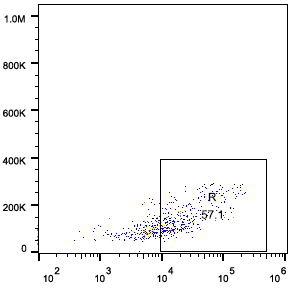

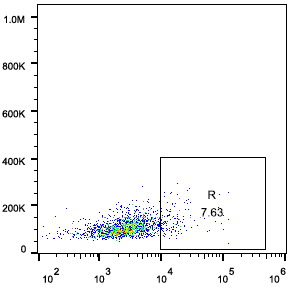

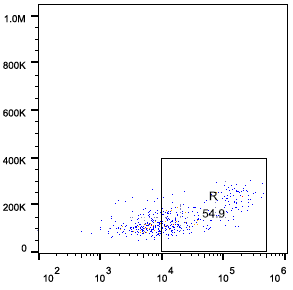

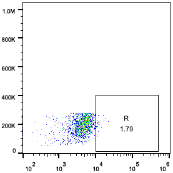

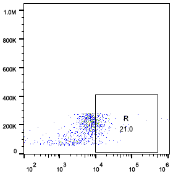


**ActRIIA^low^CD8^+^ T**

**ActRIIA^high^CD8^+^ T**


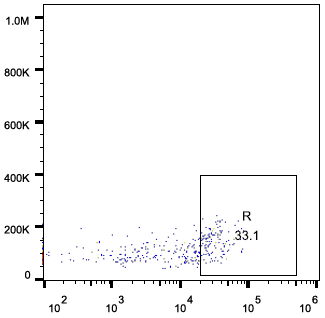

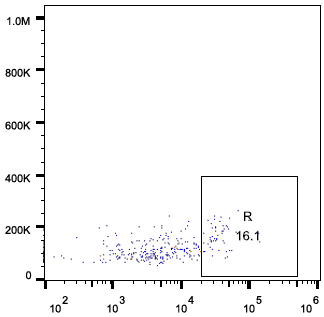

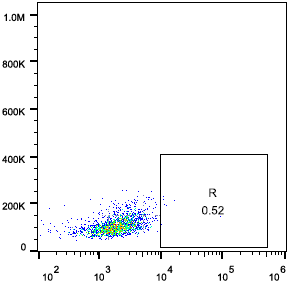

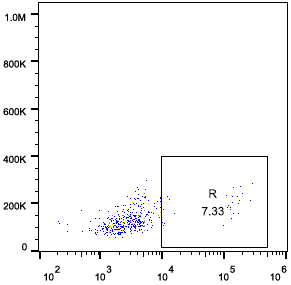

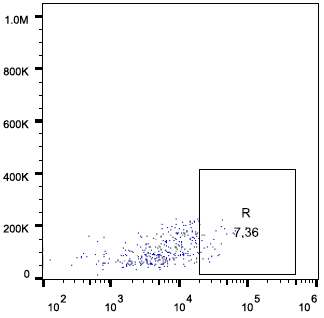

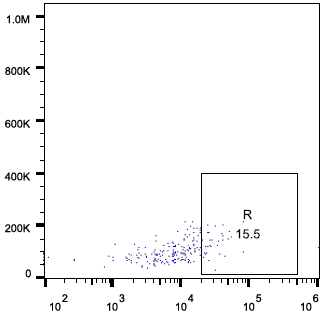


**SSC**

**CD49b CCR8 CXCR4**

**ActRIIA^low^CD8^+^ T**

**ActRIIA^high^CD8^+^ T**

**CXCR3 Ki67**

**SSC**


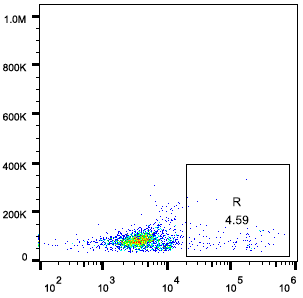

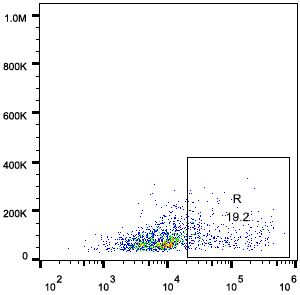

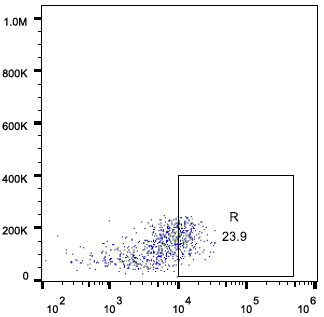

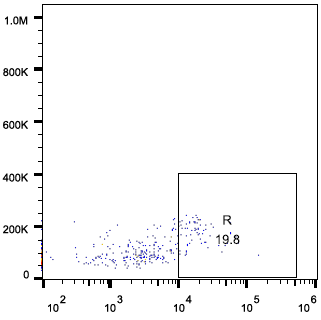


**ActRIIA^low^CD8^+^ T**

**ActRIIA^high^CD8^+^ T**

Supplemental figure 4: The expression distribution of immune checkpoints gene in ActRIIA high and ActRIIA low tumor tissues.

The correlation between ACVR2A expression and immune-infiltrating cells was calculated via CIBERSORT algorithms. The correlation between ACVR2A and immune checkpoint gene expression was calculated by Spearman’s correlation coefficient.


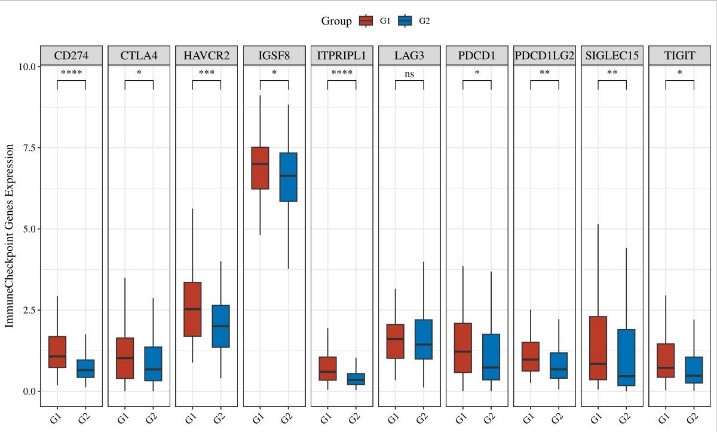

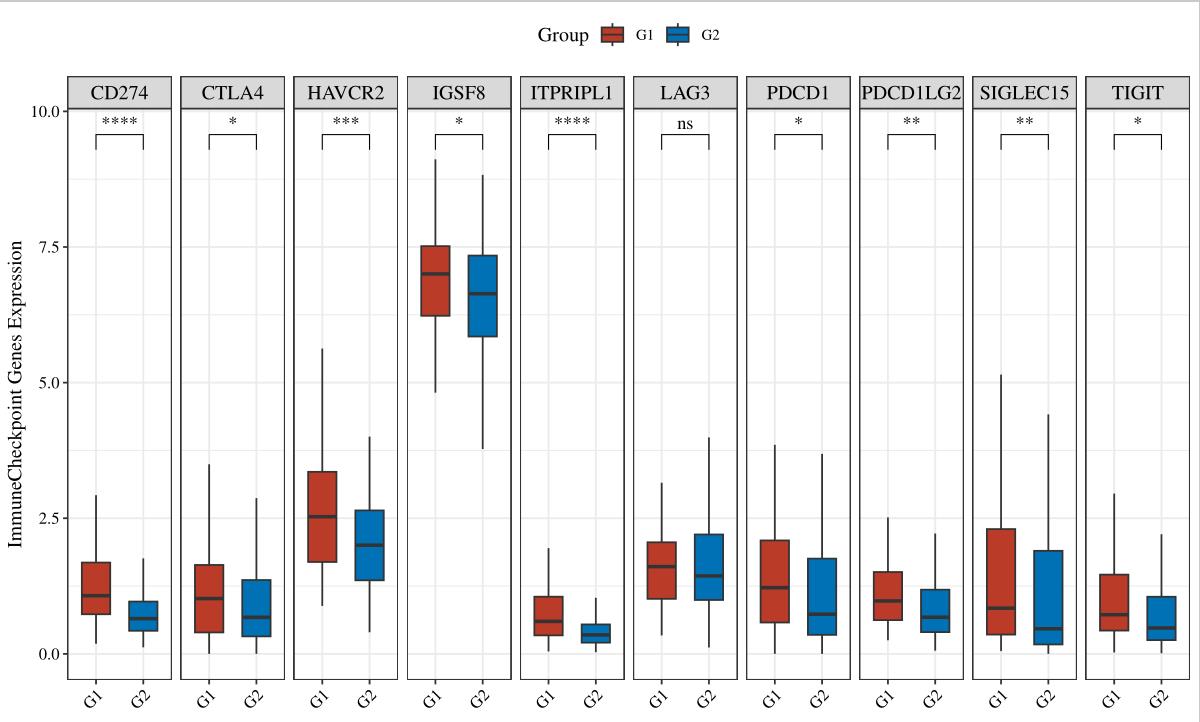


**ActRIIA^low^CD8^+^ T**


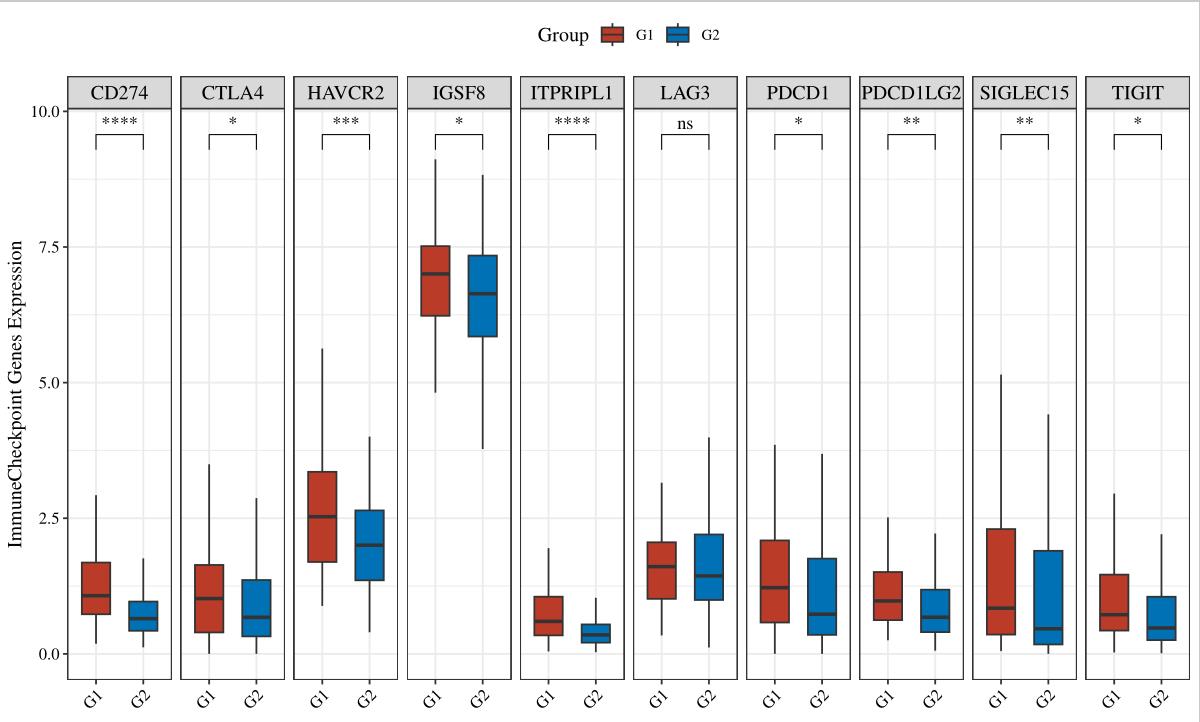


**ActRIIA^high^CD8^+^ T**

Supplemental figure 4. Correlation analysis between ACVR2A expression and immune cell infiltration. Spearman’s correlation between ACVR2A expression and immune cell infiltration in HCC. (XCELL algorithm). *p < 0.05, **p < 0.01,***p < 0.001, asterisks (*) stand for significance levels. The statistical difference of two groups was compared through the Wilcox test.
